# Preoperative localization of pulmonary nodules: virtual bronchoscopic navigation vs a 4‑hook localization needle

**DOI:** 10.20452/wiitm.2025.17930

**Published:** 2025-01-15

**Authors:** Xiaofeng Li, Xuepeng Bai, Li Xu, Jiankun Zhu, Fan Zhang, Changming Shen, Feng Jin, Yunzeng Zhang

**Affiliations:** Department of Thoracic Surgery, Shandong Public Health Clinical Center, Shandong University, Shandong, China; Department of Respiratory Endoscopy, Shandong Public Health Clinical Center, Shandong University, Shandong, China

**Keywords:** 4‑hook localization needle, localization, pulmonary nodules, virtual bronchoscopic navigation

## Abstract

**INTRODUCTION:**

Both virtual bronchoscopic navigation (VBN) and puncture with a 4‑hook localization needle are viable methods for localizing pulmonary nodules. However, there is a paucity of research that compares these 2 approaches.

**AIM:**

This study aimed to assess and compare the efficacy of and complications associated with these 2 approaches to pulmonary nodule localization.

**MATERIALS AND METHODS:**

We analyzed 223 patients who underwent VBN (n = 98) or needle localization (n = 125) of pulmonary nodules between April 2020 and December 2022. Each study group was divided into 2 subgroups, namely the solitary‑nodule group and the 2‑nodule group. We collected and analyzed data on localization time, accuracy, success rate, and complications in each group.

**RESULTS:**

In the solitary‑nodule subgroup, the mean (SD) distance between the localization point and the pulmonary nodule was 6.2 (6.1) mm for the needle‑localization group and 8.6 (4.8) mm for the VBN‑localization group (P = 0.01). In the 2‑nodule subgroup, the mean (SD) distance did not significantly differ and amounted to 8.7 (4.6) mm for the needle‑localization group and 8.4 (4.4) mm for the VBN‑localization group. However, the mean (SD) time required for localization was shorter in the VBN‑localization group (17.2 [2.6] min) than in the needle‑localization group (26.6 [3.9] min; P <0.001), which indicated that VBN was more efficient in 2‑nodule localization. The solitary nodule– and 2‑nodule–localization procedures differed significantly in terms of complications, such as pneumothorax and bleeding, with fewer complications reported in the VBN‑localization group.

**CONCLUSIONS:**

In comparison with needle localization, VBN localization was associated with fewer complications. In the case of 2 pulmonary nodules, VBN localization outperformed the needle approach, with shorter localization time, fewer complications, and no radiation exposure.

## INTRODUCTION

With the increasing availability of high‑resolution computed tomography (CT), the detection of pulmonary nodules has become more common. Consequently, surgical intervention may be necessary for specific nodules.^1^ Video‑assisted thoracoscopic surgery (VATS) has gained broad attention in the treatment of pulmonary nodules due to its significant advantages in minimally invasive procedures.

Localization procedures are particularly beneficial for subcentimeter nodules, nodules located far from the visceral pleura, ground‑glass opacities (GGOs), and nodules that are difficult to visualize using VATS.^2^ Common methods for preoperative nodule localization in clinical practice include the use of location hooks, microcoils, and iodine‑based agents. However, these methods have their limitations and may be associated with complications, such as pneumothorax and hemothorax.^3^ Currently, the most widely employed technique in CT‑guided localization is the hook‑wire system, which features a high success rate, reasonable cost, and ease of use. Of note, this method is also associated with a relatively high incidence of com‑ plications.3 In 2020, Fan et al^4^ introduced a nov‑ el pulmonary nodule localization needle known as the 4‑hook localization needle. This needle has been shown to have satisfactory efficacy in nod‑ ule localization and low risk of complications.^5^^,^^6^

**FIGURE 1:**
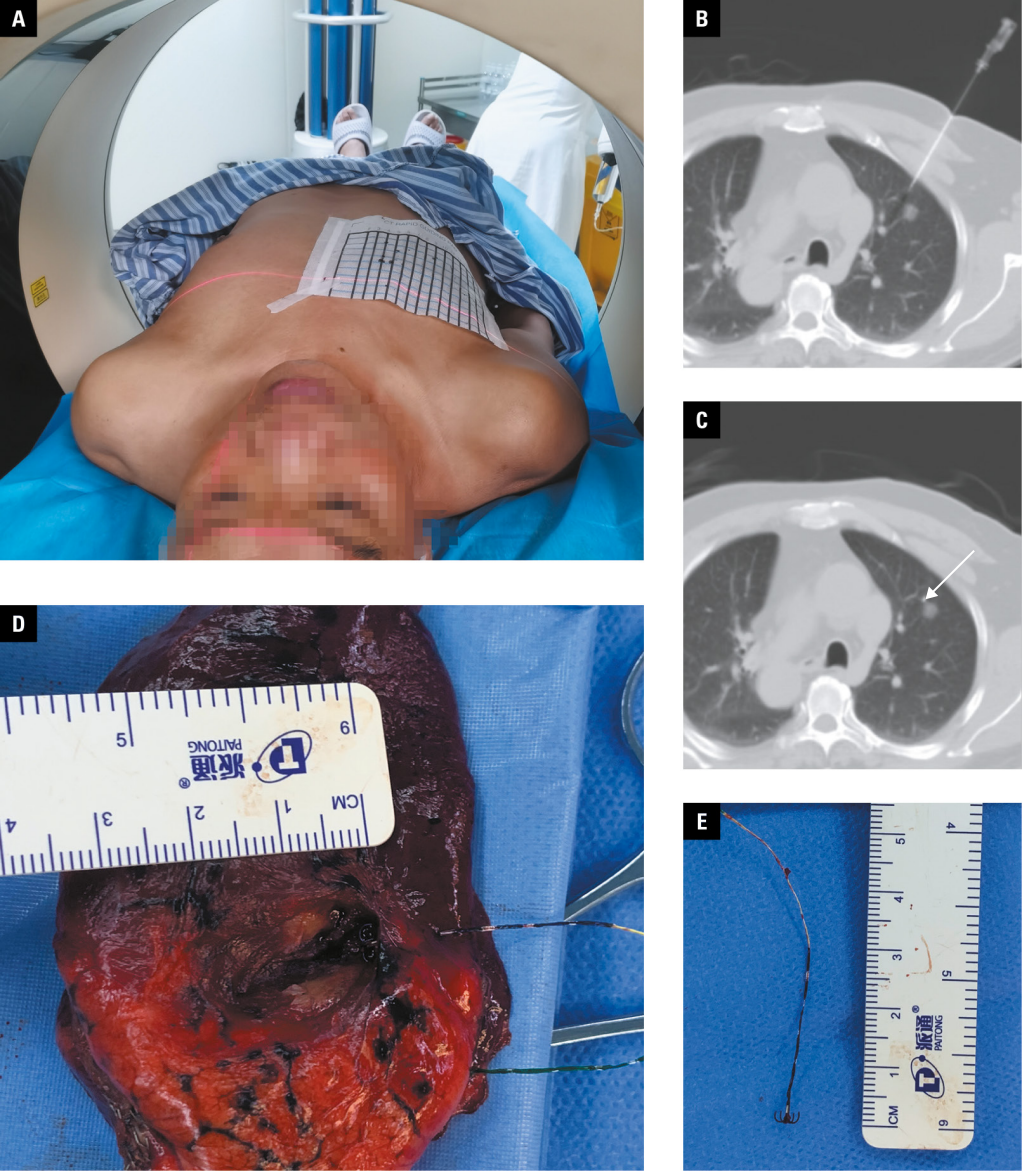
Pulmonary nodule localization using a 4‑hook localization needle; A – coronal localization by computed tomography and sagittal marking with steel needles; B – insertion of the localization needle into the lung tissue and positioning it near the pulmonary nodule selected for resection; C – release of the localization needle (arrow) and recording its relative position to the pulmonary nodule; D – resection of the needle and the surrounding lung tissue based on the markings of the localization needle and identification of the pulmonary nodule; E – removal of

Pulmonary nodule localization using a 4‑hook localization needle; A – coronal localization by computed tomography and sagittal marking with steel needles; B – insertion of the localization needle into the lung tissue and positioning it near the pulmonary nodule selected for resection; C – release of the localization needle (arrow) and recording its relative position to the pulmonary nodule; D – resection of the needle and the surrounding lung tissue based on the markings of the localization needle and identification of the pulmonary nodule; E – removal of the localization needle and checking its integrity

Injection of dyes, such as methylene blue and indocyanine green (ICG), was found to be both safe and effective.^7^^,^^8^ A bronchoscopic injection technique using ICG was reported to provide a safe and readily detectable fluorescence‑based marker in thoracoscopic surgery. This technique allows for simultaneous marking of multiple lesions and reduces the risk of complications, such as pneumothorax.^9^

As a precise modality of tumor identification, virtual bronchoscopic navigation (VBN) could assist surgeons in locating pulmonary nodules, offering a safe and accurate approach.^10^ The new generation LungPro whole‑lung navigation system (registered as the Archimedes System outside of China) enables both intra and extrabronchial navigation, which enhances its accurate vessel recognition capabilities. This navigation system enables safer and more accurate localization of target pulmonary nodules as compared with traditional bronchoscopy.^11^^,^^12^

In this retrospective study, we compared 2 pre‑ operative lung nodule localization methods: Lung‑ Pro VBN and CT‑guided percutaneous lung puncture with a 4‑hook needle.

## AIM

The aim of this study was to assess and compare the efficacy of VBN and a 4‑hook posi‑ tioning needle in locating pulmonary nodules, and to identify possible complications associated with their use.

**FIGURE 2:**
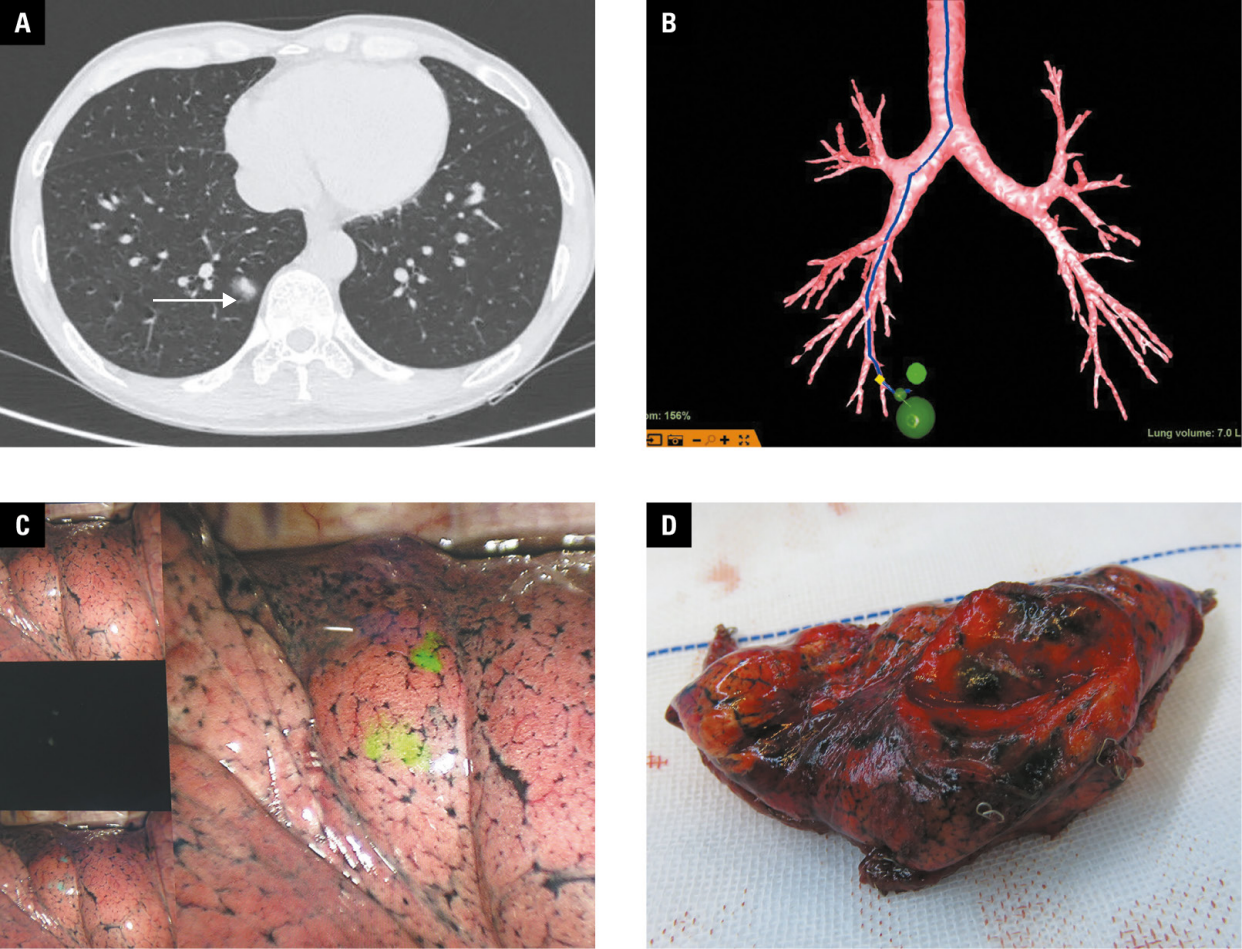
Pulmonary nodule localization using virtual bronchoscopic navigation (VBN); A – computed tomography image showing a small pulmonary nodule (arrow) located in the right lower lobe; B – preoperative design of the VBN localization path with the LungPro software; C – injection of a indocyanine green marker into the corresponding site based on the virtual navigation pathway; D – verification of localization accuracy after thoracoscopic surgery

## MATERIALS AND METHODS

We conducted a retrospective analysis of patients with pulmonary GGOs who underwent preoperative nodule localization prior to single‑port thoracoscopic surgery. The patients were divided into 2 groups based on the preoperative localization method: in one group, the lung nodules were localized using the ICG injection method guided by LungPro (Broncus Medical, Inc., San Jose, California, United States) VBN (hereinafter referred to as VBN localization), while in the other group, percutaneous lung puncture with a 4‑hook localization needle was performed (hereinafter referred to as needle localization). The study groups were then further divided into the solitary‑nodule localization subgroup and the 2‑nodule localization sub‑group, based on the number of localized nodules. Demographic characteristics, including age, sex, height, and weight, were recorded.

In the needle‑localization group, the localization procedure was chosen based on preoperative imaging data and the surgical approach. Localization markers were injected and CT scans performed to determine both the position and depth of needle insertion. Sterile drapes were used after routine disinfection. Local infiltration anesthesia was administered with 2% lidocaine. CT imaging was used to facilitate insertion and to guide the puncture needle through the chest wall and pleura, all the way to the pulmonary nodule. Once the pushing device reached the bottom of the localization needle sheath, the localization needle was released and secured to the nodule or its margin. Initially, the puncture needle was left free, and it was subsequently retracted to enable removal of the needle sheath. Eventually, the puncture needle and the pushing device were pulled out together from the patient’s body. A second CT scan was performed to position both the localization needle and the nodule. In the 2‑nodule subgroup, this process was repeated for the other nodule. The procedure was immediately followed by VATS. The steps of the localization procedure are summarized in FIGURE 1.

In the VBN‑localization group, the procedure steps were as follows FIGURE 2: prior to surgery, the patient’s chest CT data were imported into the LungPro system to maximally improve visualization of the airways surrounding the lesion. The LungPro system could confirm that the bronchi of the target or adjacent lung segments were as close to the lesion as possible. Special attention was paid to marking the location where the pleura was stained and to determining the relationship between each staining location and the lesion. After general anesthesia was induced, an 8‑mm single‑lumen endotracheal tube was inserted. The first step in the preoperative navigation plan was to assess the feasibility of the pathway using a bronchoscope with a working channel greater than 1.8 mm. A brush‑inspected outer sheath was used to inject 0.2 ml of ICG if feasibility was con‑ firmed; otherwise, a bronchoscope with an outer diameter of 2.8 mm was chosen to inject the dye. In each investigated site, 0.2 ml of the dye was injected using a 1‑ml empty needle first, and then 20 ml of air was injected at the same site using a 20‑ml empty needle at a slower rate. The spatial relationship between the staining localization points and pulmonary nodules was confirmed. The single‑lumen endotracheal tube was then replaced with a double‑lumen one, and surgical preparations were executed promptly.

**TABLE 1:** Clinical characteristics of patients undergoing localization of a solitary pulmonary nodule

Variable		Needle‑localization group (n = 84)	VBN‑localization group (n = 66)	P value
Age, y, mean (SD)		57.4 (9)	54.6 (9.8)	0.07
Sex, n	Men	49	34	0.4
	Women	35	32	
BMI, kg/m2 , mean (SD)		24.96 (3.9)	23.92 (3.11)	0.08
Nodule size, mm, mean (SD)		10.2 (3.6)	10 (2.8)	0.79
Nodule depth, mm, mean (SD)		10.8 (10)	10.1 (9.4)	0.67
Nodule density, n	pGGO	50	43	0.48
	GGN	34	23	
Nodule location, n	UL /ML	56	40	0.44
	LL	28	26	
Pathological diagnosis	Benign / AAH	21	15	0.63
	AIS	16	8	
	MIA	32	29	
	IAC	15	14	

Abbreviations: AAH, atypical adenomatous hyperplasia; AIS, adenocarcinoma in situ; BMI, body mass index; GGN, ground‑glass nodule; IAC, invasive adenocarcinoma; LL, lower lobe; MIA, minimally invasive adenocarcinoma; ML, middle lobe; pGGO, pure ground‑glass opacity; UL, upper lobe; VBN, virtual bronchoscopic navigation

**TABLE 2:** Clinical characteristics of patients undergoing localization of 2 pulmonary nodules

Variable		Needle localization group (n = 41)	VBN localization group (n = 32)	*P* value
Age, y, mean (SD)		58 (7)	58.2 (7.8)	0.88
Sex, n	Men	21	16	0.92
	Women	20	16	
BMI, kg/m^2^, mean (SD)		25.9 (3.48)	25.08 (2.49)	0.24
Nodule size, mm, mean (SD)		9.5 (2.8)	9 (3.5)	0.38
Nodule depth, mm, mean (SD)		10.7 (8)	9.5 (6.4)	0.31
Nodule density, n	pGGO	69	52	0.65
	GGN	13	12	
Nodule location, n	UL /ML	43	31	0.63
	LL	39	33	
Pathological diagnosis, n	Benign / AAH	10	11	0.89
	AIS	36	30	
	MIA	31	20	
	IAC	5	3	

Abbreviations: see TABLE 1

### Statistical analysis

The measurement data were expressed as mean (SD) and analyzed using either the independent samples t test or Mann–Whitney test. Count data were presented as percentag‑ es and analyzed using the χ^2^ test or Fisher exact test. The statistical analysis was performed us‑ ing the SPSS 22.0 software (SPSS Inc., Chicago, Illinois, United States). A P value below 0.05 was considered significant.

### Ethics

This study was reviewed and approved by the ethics committee of the Shandong Public Health Clinical Center (2021XKYYEC-14). The patients provided written informed consent to participate in the study

## RESULTS

From April 2020 to December 2022, a total of 223 patients were included in this study. The needle‑localization group consisted of 125 individuals, with 84 undergoing solitary‑nodule localization and 41 undergoing 2‑nodule localization. The VBN group comprised 98 participants, with 66 undergoing solitary‑nodule localization and 32 undergoing 2‑nodule localization. The pa‑ tient age ranged from 19 to 82 years. The demo‑ graphic and clinical characteristics of the patients undergoing localization of solitary and 2 nodules are summarized in TABLE 1 and TABLE 2, respectively. No significant differences between the 2 groups were observed in terms of age, sex, body mass index, and nodule size, location, depth, density, or pathological type.

Procedural characteristics of the patients undergoing localization of solitary nodules are presented in TABLE 3. The mean (SD) distance between the localization point and the pulmonary nodule was 6.2 (6.1) mm in the needle‑localization group, while in the VBN‑localization group it was 8.6 (4.8) mm (P = 0.01). Interestingly, the patients undergoing VBN localization experienced fewer complications, such as pneumothorax and pleural reactions, were not observed. In contrast, 20 of the 84 patients in the needle‑localization group developed complications. The rates of procedural success were comparable between the groups. In the needle‑localization group, needle dislodgement was reported in 3 patients, whereas in the VBN‑localization group, dye dispersion was noted in a single patient.

TABLE 4 presents procedural data of the patients undergoing localization of 2 pulmonary nodules. The mean (SD) distance between the nodules was 8.7 (4.6) mm in the needle‑localization group and 8.4 (4.4) mm in the VBN‑localization group. The difference was nonsignificant. The mean (SD) duration of localization of 2 nodules was 26.6 (3.9) minutes in the needle‑localization group and 17.2 (2.6) minutes in the VBN‑localization group. In the VBN‑localization group, significantly less time was required to perform the procedure. 

**TABLE 3:** Procedural characteristics of patients undergoing localization of a solitary pulmonary nodule

Variables		Needle‑localization group (n = 84)	VBN‑localization group (n = 66)	P value
Distance between the localization point and the pulmonary nodule, mm, mean (SD)		6.2 (6.1)	8.6 (4.8)	0.01
Localization time, min, mean (SD)		12.5 (2.5)	13.2 (2.4)	0.06
Complications, n	Absent	64	65	<0.001
	Present	20	1	–
Complication type, n	Asymptomatic pneumothorax	10	0	
	Symptomatic pneumothorax	3	0	
	Pulmonary hemorrhage	4	1	
	Bleeding and pneumothorax	1	0	
	Pleural reaction	2	0	
Successful localization rate		81/84^a^	65/66b	0.93

a Three cases of needle dislodgement

b One case of dye dispersion

Abbreviations: see TABLE 1

**TABLE 4:** Procedural characteristics of patients undergoing localization of 2 pulmonary nodules

Complications, n	Absent	64	65	<0.001
	Present	20	1	
Complication type, n	Asymptomatic pneumothorax	10	0	–
	Symptomatic pneumothorax	3	0	
	Pulmonary hemorrhage	4	1	
	Bleeding and pneumothorax	1	0	
	Pleural reaction	2	0	
Successful localization rate	81/84a	65/66b	0.93	

a One case of needle dislodgement, 2 cases of failed localization after pneumothorax

b One case of dye dispersion

Abbreviations: see TABLE 1

No significant complications were observed among the 32 patients in the VBN‑localization group. However, among the 41 patients in the needle‑localization group, 13 developed com‑ plications. The intergroup difference was significant. In the needle‑localization group, a single case of decoupling and 2 cases of incomplete localization of the second nodule due to pneumothorax were reported. In contrast, in the VBN‑ ‑localization group, only 1 case of dye dispersion was observed. The difference in the procedural success rate was not significant.

## DISCUSSION

VATS is a standard surgical approach for the treatment of pulmonary nodules.^13^ However, locating small, deep, subsolid lesions or lesions presenting as GGOs on CT scans can be challenging during surgery, as they do not manifest as distinguishable changes on the adjacent visceral pleural surface. Furthermore, the restrict‑ ed access through small incisions makes manual detection even more difficult.

Various techniques are adopted for lesion localization, including hook wires, microcoil, dye, iodinated contrast agents, radioactive markers, and fiducial markers. Among these, CT‑guided hook‑wire localization is the oldest and most commonly used method. Its advantages comprise a high success rate, accelerated localization, simplicity, low cost, and intraoperative visualization. However, it also has certain limitations. Surgery cannot be initiated immediately after localization, as the patient needs to be transferred from a CT suite to an operating room. Additionally, the hook wire remains outside the patient’s body before insertion, causing discomfort. A meta‑analysis of 46 clinical studies^3^ reported a high incidence of complications with hook‑wire localization, including pneumothorax (35%) and bleeding (16%). In order to address the aforementioned limitations, the localization hooks have been continuously improved. In a study by Fan et al,^4^ a novel type of lung nodule localization needle, called a 4‑hook localization needle, was described. This innovative needle design differs from that of traditional hook wires, as it features a claw with 4 hooks at the front end, each measuring 4 mm in length and made of a nickel–titanium shape‑memory alloy. When deployed, these hooks expand to a diameter of 5 mm. The back end of the needle is equipped with an 86‑mm marker thread. Studies demonstrated that the 4‑hook needle exhibited superior performance in terms of safety and effectiveness, and a reduced incidence of complications and dislodgement.^4^^,^^14^ Additionally, the the 4‑hook needle demonstrated shorter procedure times, more precise localization, greater user‑friendliness, and reduced operator requirements, as compared with traditional coil and hook‑needle localization methods.^5^^,^^6^^,^^15^^,^^16^ In addition to preoperative CT‑guided localization, there have been significant advancements in the preoperative aspects of bronchoscopy. A meta‑analysis^17^ suggested that bronchoscopic mark‑ ing was a highly effective and safe preoperative marking technique for lung resection, making it the preferred technology in thoracic surgery. The 2 currently popular positioning methods include electromagnetic navigation bronchoscopy and VBN. As electromagnetic navigation is cost‑ ly, VBN is easier to implement. It involves using virtual bronchoscopic images to guide the bron‑ choscope to surrounding lesions. By combining an automated system that searches for the bronchial pathway to the target, virtual bronchoscopic images of the bronchial pathway can be generated and displayed simultaneously with the real bronchoscopic images. This technique can serve as a marker in thoracoscopic surgery. VBN is an effective method for bronchoscopic examination of peripheral lesions.^18^ It can be used alone or in combination with other bronchoscopic techniques to guide the bronchoscope to the target lesion. VBN is a safe, painless, and cost‑effective approach, which makes it suitable for preoperative localization of pulmonary nodules.^19^

When selecting tracers and marking techniques, several factors need to be considered, including the depth of nodules in the lung parenchyma, tracer properties, and the time interval between marking and surgery.^20^ ICG fluorescence has been found to be valuable during VATS due to its ability to penetrate deep tissues.^21^^,^^22^ Near‑infrared fluorescence thoracoscopy can detect the presence of ICG up to a depth of 24 mm. Visibility can be significantly improved by injecting ICG markers during virtual bronchoscopy and combining it with near‑infrared fluorescence imaging. In the context of small lesions that are not easily perceptible, the ability to visualize the lesion during surgery is essential for its accurate localization.^23^^,^^24^^,^^25^^,^^26^

In the VBN‑localization group, the LungPro navigation system, OptoMedic fluorescence tho‑ racoscope, and bronchoscope were used to pre‑ plan the target sites and pathways. Prior to surgery, in the operating room, 0.2 ml of ICG were injected into each target site. Previous studies indicated that the fluorescent spot formed by 0.1 ml of ICG had a mean (SD) size of 10.4 (2.2) mm.^23^ However, during the procedure, a portion of the dye remained on the catheter wall and did not enter the lung tissue. Of note, ICG diffuses rapidly in the lung tissue, which emphasizes the need for prompt surgery after puncture localization.^7^ A majority of previous studies have focused on solitary‑nodule localization. In contrast, our study aimed to compare the advantages and disadvantages of both solitary and 2‑nodule localization. We noted a high success rate for both needle positioning and VBN positioning, with no significant differences between the single‑shot and

double‑shot positioning methods.

Notably, decoupling was the main cause of positioning pin failure, which was significantly improved after the 4‑hook technique was implemented. Another reason for localization failure was the considerable distance between the localizing point and target nodule resulting from the proximity of the nodule to the mediastinal surface or the top of the chest. Additionally, pneumothorax caused by multiple punctures had an impact on the success rate of further localization. VBN localization failure was primarily attributable to dye diffusion. Overall, both methods demonstrated a high success rate in our study. They are accurate in locating pulmonary nodules with the use of imaging techniques. With appropriate navigation guidance, they can effectively locate lung nodules. The success rate of VBN localization depends on the technology and equipment involved, as well as the level of surgical skill and operational experience.

In comparison with VBN, solitary nodule localization with a needle appears more precise. The needle localization technique involves releasing the hooks after the needle position is confirmed on CT, which allows for fine adjustments and theoretically higher accuracy. However, this may increase the occurrence of complications. Previous reports indicated that a higher frequency of puncture may increase the risk of pneumothorax, including iatrogenic pneumothorax.^27^

In the context of 2‑nodule localization, no significant improvement in accuracy was observed for the needle localization technique, as com‑ pared with VBN localization. This lack of advantage may be attributed to the fact that patients experienced pneumothorax or cough‑related dis‑ comfort after the initial localization and subsequently presented a lower level of cooperation. However, both solitary nodule and 2‑nodule localization techniques demonstrated fewer complications with VBN, which indicated a clear advantage of this modality. These findings are consistent with the results reported elsewhere.^9^ The primary reason for this advantage is that VBN localization does not involve puncturing the chest wall and pleura, and access is secured through the natural bronchial airways, thereby reducing the risk of complications. In our study, we used a 4‑hook needle for localization in 84 patients with a solitary nodule and observed a complica‑ tion rate of 23.8%. In comparison, Fan et al^4^ and Chen et al^16^ reported complication rates of 15% and 37%, respectively. The difference in complication rates may be due to the subjectivity in how a complication was defined and whether there was a distinction between solitary and multiple‑nodule localization.

Our results indicated that VBN was superior to lung puncture in the preoperative localization of pulmonary nodules, especially in the 2‑nodule cases. This technique requires less time and has a lower complication rate but, at the same time, shows a similar success rate and accuracy as needle localization. The VBN technique is particularly suitable for patients with emphysematous lungs or those requiring localization of multiple or bilateral nodules. However, traditional bronchoscopic localization has lower accuracy. Of note, CT‑guided labeling shows better out‑ comes for nodules smaller than 12 mm, while bronchoscopic labeling yields better results for deeper nodules.^20^ Liquid markers need not be removed along with pulmonary nodules as they can be absorbed by the body. Therefore, the VBN targeting technique allows for the preservation of a greater amount of lung tissue. ICG tends to diffuse easily, and its short retention time can be further improved to make it longer. During needle positioning, the lung tissue surrounding the needle must be excised, the body is ex‑ posed to radiation, and some amount of the lung tissue needs be removed to allow the needle to pass through. The advantages of needle localization include preoperative positioning and easier identification during surgery.

The VBN technique offers several advantages over lung puncture in localizing mediastinal nodules. It allows for locating a wider range of nodules while causing fewer complications. Moreover, it may perform even better in locating multiple nodules. However, there are some other factors that should be considered. First, the use of VBN is limited by specific technical and equipment conditions, such as virtual navigation systems, bronchoscopes, and computer reconstruction software. This imposes strict requirements on the resources and economic costs for medical institutions. Second, physicians per‑ forming VBN need to be adequately experienced and proficient to guarantee accuracy and safe‑ ty. Therefore, medical institutions need to assess whether they are prepared to implement the VBN technique considering both equipment and human factors.

Limitations The present study has certain limitations, such as a relatively small sample size, which may cause a selection bias. The localization of 3 or more pulmonary nodules was not analyzed in this study. Sample upsizing could provide a more comprehensive evaluation of the comparative results between the 2 localization methods. Additionally, it is important to acknowledge that this study may have some hidden biases, and we cannot completely exclude the interference of other potential factors.

## CONCLUSIONS

In the preoperative localization of pulmonary nodules, VBN localization was found to have a slightly lower accuracy in locating a solitary pulmonary nodule, as compared with needle localization. However, this slight decrease in accuracy had no impact on the successful resection of nodules. On the other hand, VBN localization was associated with fewer complications. When used for locating 2 pulmonary nodules, the VBN technique performs well, with shorter localization time, fewer complications, and no radiation exposure. Taken together, VBN is a feasible, safe, and efficient technology for the localization of pulmonary nodules, especially in the 2‑nodule cases.
